# Animal diversity and ecosystem functioning in dynamic food webs

**DOI:** 10.1038/ncomms12718

**Published:** 2016-10-05

**Authors:** Florian D. Schneider, Ulrich Brose, Björn C. Rall, Christian Guill

**Affiliations:** 1 Institut des Sciences de l'Evolution (ISEM), Université Montpellier, CNRS, IRD, UMR 5554, C.C.065, 34095 Montpellier Cedex 05, France; 2 Senckenberg Biodiversity and Climate Research Centre (BiK-F), Senckenberganlage 25, 60325 Frankfurt am Main, Germany; 3 German Centre for Integrative Biodiversity Research (iDiv) Halle-Jena-Leipzig, Deutscher, Platz 5e, 04103 Leipzig, Germany; 4 Institute of Ecology, Friedrich Schiller Universtiy Jena, Dornburger-Strasse 159, 07743 Jena, Germany; 5 Institute for Biodiversity and Ecosystem Dynamics, University of Amsterdam, PO Box, 94248, 1090 GE Amsterdam, The Netherlands; 6 Institute of Biochemistry and Biology, University of Potsdam, Maulbeerallee 2, 14469 Potsdam, Germany

## Abstract

Species diversity is changing globally and locally, but the complexity of ecological communities hampers a general understanding of the consequences of animal species loss on ecosystem functioning. High animal diversity increases complementarity of herbivores but also increases feeding rates within the consumer guild. Depending on the balance of these counteracting mechanisms, species-rich animal communities may put plants under top-down control or may release them from grazing pressure. Using a dynamic food-web model with body-mass constraints, we simulate ecosystem functions of 20,000 communities of varying animal diversity. We show that diverse animal communities accumulate more biomass and are more exploitative on plants, despite their higher rates of intra-guild predation. However, they do not reduce plant biomass because the communities are composed of larger, and thus energetically more efficient, plant and animal species. This plasticity of community body-size structure reconciles the debate on the consequences of animal species loss for primary productivity.

## Abstract (web summary)

Losing animals from food webs could reduce ecosystem function, but drivers of this pattern are difficult to disentangle. With food web simulations, Schneider *et al*. show that high animal diversity does not release plants from top-down control owing to a balancing effect of increased animal body size.

## Graphical abstract

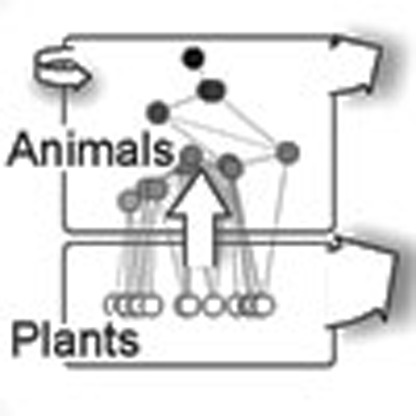

Although there is much evidence that ecosystem functioning is a product of organismal activity^1^, the relationship between species diversity and ecosystem functioning remains enigmatic. Alarmed by the recent rates of species extinction across all ecosystems, much ecological research over the past two decades has been driven by the biodiversity effects on the magnitude and stability of ecosystem functions (the biodiversity-ecosystem functioning debate)^1^,^2^,^3^. While most of this research focused on variation in plant diversity^4^,^5^, fewer studies addressed the consequences of declining animal diversity^6^,^7^,^8^ despite the higher extinction risk at higher trophic levels^9^. Consequently, we lack a generalized understanding of the relationship between animal diversity and ecosystem functioning: Apparently idiosyncratic, positive as well as negative consequences for plant primary productivity in response to animal species loss from multi-trophic communities have been observed^10^,^11^,^12^,^13^,^14^, which cannot be explained by species richness alone.

Partially, this dichotomy of effects may be explained by the ecosystem's balance between niche complementarity effects and community trophic cascades^15^,^16^,^17^. A simply structured animal community of a single trophic level is limited to cause direct negative effects on the trophic level below. Therefore, it would become more exploitative on the plant community, due to niche complementarity^18^, as animal diversity increases. This mechanism is highly relevant when horizontal diversity, that is, diversity within a trophic level increases^8^. For instance, an increase in the number of obligate herbivorous species should result in higher absolute rates of herbivory. In contrast, predation among animals can generate indirect positive effects on the plant community: the predominant consumption of animal prey releases the basal community from top–down pressure due to trophic cascades. As vertical diversity increases through added trophic levels, the animal communities' total effect on the basal plant community would be reduced^8^,^10^,^15^,^19^,^20^. Accordingly, the net effect of increasing animal diversity on basal productivity could be negative or positive depending on the relative dominance of increased niche complementarity or trophic cascades, respectively^8^.

In natural ecosystems, however, vertical and horizontal diversity are not independent of each other but are linked due to the complex feeding interactions within the animal community. As species number increases, animals cover larger parts of the resource niche space (horizontal diversity) and occupy new trophic levels (vertical diversity)^8^,^21^,^22^. Generalist feeding leads to omnivory (that is, feeding on resources across trophic levels) and causes intraguild predation (that is, feeding on resources of the same trophic group) to be more common in diverse food webs^23^,^24^. Consequently, any clear distinction between trophic levels would be lost^8^, which has been hypothesized to make trophic cascades less likely^7^,^10^,^18^,^19^,^20^,^25^.

These counteracting mechanisms inhibit any generalized predictions about how animal diversity affects the plant compartment and the processes and ecosystem functions related to it^6^,^16^. Usually in experimental studies, the plant biomass standing stock, rates of herbivore consumption and metabolism are assessed to quantify ecosystem functions. Therefore, in this study we employ dynamic simulations of complex food webs to assess general patterns in these community level quantities of ecosystem function in response to changes in animal diversity (Fig. 1).

Food-web models can scale individual and population level mechanisms to complex communities of interacting species^16^ to make predictions about the consequences of altered diversity across trophic levels^26^,^27^,^28^,^29^. In previous applications, however, species richness within a trophic level (horizontal diversity) and the number of trophic levels (vertical diversity) were investigated separately^3^,^16^, with only few exceptions^28^,^30^. None of these models reflected the variability and complexity of natural food webs, which differ strongly in species richness and composition as well as the number and strength of their interactions. We fill this gap by extrapolating dynamic models based on allometric constraints^31^, that have successfully been applied to predict population dynamics and consequences of species extinctions in simple modules, to the context of entire food webs^32^,^33^,^34^.

Allometric, or body mass, constraints on multiple species traits like movement speed, reproduction rates, volume-to-surface ratios and metabolic rates have long been appreciated^35^. More recently, the implications of allometry for community level properties, such as feeding rates^36^,^37^,^38^,^39^,^40^, niche differentiation^32^,^38^,^41^,^42^ and food web structure^23^,^41^,^43^,^44^, have been quantified. Therefore, we apply an allometric model that defines the consumer (that is, animal) species' feeding rates on its resource (that is, plants and other animals) as functions of consumer and resource body masses (‘allometric functional response')^31^,^32^,^36^,^37^,^38^,^39^. The potential feeding rate is highest for an energetically optimal resource size, while smaller and larger resource species are less efficiently foraged for (Fig. 2, Methods section).

During the simulations, biomasses of species adjust dynamically and species extinctions occurr before a steady state is achieved. Thus, the species that comprise the final community were ‘selected' by energetic processes among the allometrically defined species. While similarly sized species exploit the same resources and are vulnerable to the same consumers, which synchronizes their population dynamics, differently sized species contribute complementary features to the community, for example, exploit a resource that cannot be accessed by others. By occupying a spot in the upper range of our model's niche axis, particularly large animals would form a predatory trophic level on top of the primary consumer community. The observed levels of biomass of the animal and plant community as well as the process rates and energetic losses on the community level arise implicitly from the allometric constraints at the population level. Being based on well understood mechanisms at the population level, this is a generic approach to the functions provided at the ecosystem level^45^ (Fig. 1).

Using this generic model framework, we investigate whether increasing animal diversity causes stronger top–down control on the plant community via enhanced direct feeding interactions and complementarity effects, or if it rather weakens top–down control due to the increase of intraguild predation among animals. We observe that despite increased intraguild predation in diverse animal communities, plants are not released from top–down pressure due to the plasticity of community size structure.

## Results

### Dynamic food-web model

We cast the concepts outlined above into a unifying ecological framework by applying a network-theoretic approach assuming that the species (nodes), connected by dynamic feeding interactions (edges), compose the higher level characteristics of ecosystems^3^,^14^,^16^,^18^. The potential feeding rate curves of all animal species thus define the network structure of the entire community. A consumer feeds on all species present in the local food web that are within a certain body-mass range, including other consumers (Fig. 3a,b)^41^. By this definition, similarly sized species are redundant (as species 13 and 14 in Fig. 3a,b), while differently sized species are complementary (as species 11 and 12 in Fig. 3a,b). Also, larger predators occupy a higher trophic position in the food web which forms distinct trophic levels and induces cascading effects^46^. We believe that this model significantly reflects the major part of bioenergetic fluxes in ecosystems where body size is the dominant constraint on feeding rates and food web structure, such as marine and freshwater systems or terrestrial below-ground systems^38^,^39^,^41^,^44^.

We applied the model to simulate population dynamics of 21,461 randomly sampled communities over a large gradient of animal species richness until a steady state was reached. We included 10–100 animal species of different body mass on top of 30 plant species to build plausible communities of variable vertical and horizontal diversity (Fig. 3c).

### Ecosystem functions respond to animal diversity

**Figure 1:**
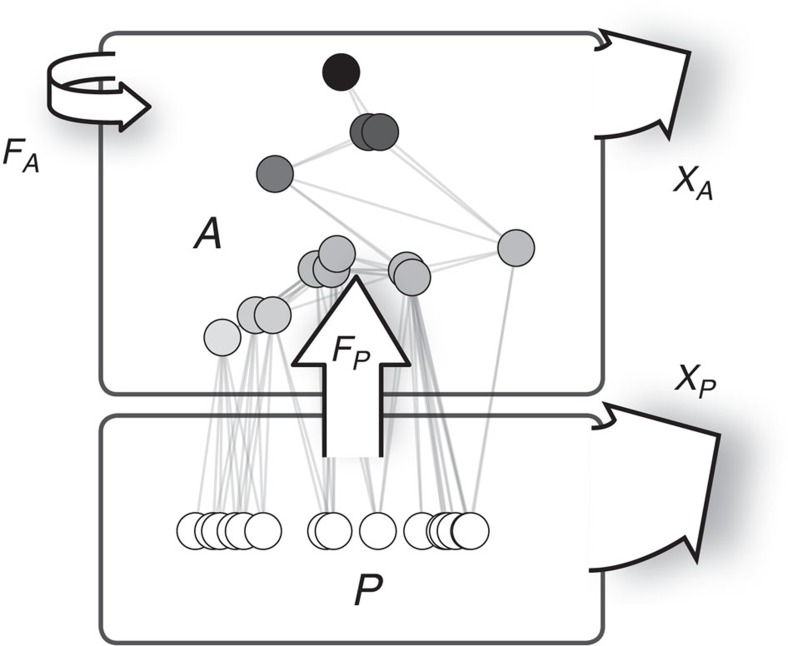
Schematic diagram of the ecosystem model. The animal community, *A*, feeds on the plant community, *P*, with rate *F*_*P*_, but also on members of the own consumer guild with rate *F*_*A*_. Both, plant and animal community, lose energy due to metabolic demands, *X*_*P*_ and *X*_*A*_, respectively.

We investigated how increasing animal diversity affects ecosystem functioning, defined as the summed biomass stocks of plant and animal species (*P* and *A*, respectively), the rates of consumption on the plant community (*F*_*P*_) and within the animal community (that is, intraguild predation, *F*_*A*_), as well as the energetic losses due to metabolism of both compartments (*X*_*P*_ and *X*_*A*_; Fig. 1). We found that as the number of animal species increased, the total biomass of animals, *A*, increased as well (Fig. 4a; *a*=0.35, and *α*=1.2 as least squares estimates of power-law relationship of the shape 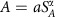 fitted as a linear model on log–log transformed data; Residual standard error on degrees of freedom: s.e.*=*0.012; Goodness of fit as indicated by Coefficient of determination: *R*^2^=0.31). In contrast, the total biomass of plants, *P*, stagnated (Fig. 4b; *a*=29.75, and *α*=−0.08, s.e.=0.005, *R*^2^=0.01). Along with the increase in animal biomasses, we observed an increase in intraguild predation rates, *F*_*A*_ (Fig. 4c, *a*=0.004, and *α*=0.92, s.e.=0.012, *R*^2^=0.2). Despite the stable total biomass of plants, we found an increase in the consumption of plants by animals, *F*_*P*_ (Fig. 4d; *a*=0.1 and *α*=0.61, s.e.=0.005, *R*^2^=0.38). Moreover, with increasing animal species richness the total animal metabolic rates, *X*_*A*_, increased (Fig. 4e; *a*=0.05 and *α*=0.58, s.e.=0.005, *R*^2^=0.38) while the total metabolic rates of plants, *X*_*P*_, decreased (Fig. 4f; *a*=6.72 and *α*=−0.54, s.e.=0.006, *R*^2^=0.3).

### Sensitivity to model parameters

For the patterns described above, we explored the sensitivity to parameter choice. The majority of the parameters were randomly drawn from normal distributions (allometric scaling parameters, hill-exponent of functional response, predator interference) and mostly had no visible effects on the relationship between animal diversity and ecosystem functions. Solely the allometric scaling exponent of consumer body mass in attack rates, *β*_*i*_, had important leverage on the effect of species richness on plant biomass: low exponents resulted in positive effects and high exponents resulted in negative effects of animal species richness on plant biomass, but left other ecosystem functions unaffected (Supplementary Figs 1–6, Supplementary Methods). While higher plant species richness (*S*_*P*_=50) did not alter the observed patterns qualitatively, the total plant biomass in communities with lower plant species richness (*S*_*P*_=10) responded negatively to animal species richness along with a stronger reduction in plant respiration (Supplementary Fig. 7, Supplementary Methods). To assess the role of omnivore feeding as opposed to strict herbivory, we ran an alternative simulation where 50% of the animals were only consuming plants. This case of a more pronounced trophic structure strengthened the suppression of plant biomass with increasing diversity via increasing plant respiration (Supplementary Fig. 8, Supplementary Methods). Lower or higher rates of nutrient turnover did not influence the relationship qualitatively (Supplementary Fig. 9, Supplementary Methods), but had an effect on the quantity of biomasses, rates and losses, indicating that bottom–up control was left unimpaired by animal diversity. We conclude that the structural features of the model, rather than the precise parameter values, are responsible for the observed patterns.

### Plasticity of community size structure

The dynamic reallocation of biomass within the animal and plant community towards larger species was responsible for the decoupling of feeding rates and plant biomass. This biomass shift translates into larger average individual body masses of animals as species richness increased (Fig. 5a; *a*=187.91, and *α*=0.13, s.e.=0.011, *R*^2^=0.01). On the basal trophic level, larger plants were favoured over smaller plants, and this effectively reduced total metabolic losses while allowing plant biomass to remain constant (Fig. 5b; *a*=0.75 and *α*=0.48, s.e.=0.013, *R*^2^=0.06).

In summary, with increasing animal species richness and an accumulation of more biomass in the animal community (Fig. 6, compartment *A*) the total biomass of plants could nevertheless be maintained at the same level (Fig. 6, compartment *P*). The animals' consumption of plants (Fig. 6, rate *F*_*P*_) increased, which enhanced the metabolic rate in the animal community (Fig. 6, compartment *A*, rates *F*_*P*_ and *X*_*A*_). In the plant community, the increased loss of biomass to consumption was compensated by a reduction in community metabolism (*X*_*P*_), which rendered the plant community more efficient in maintaining biomass.

## Discussion

We applied a dynamic simulation approach to investigate the relationship between animal diversity and ecosystem functioning represented by standing stocks of biomasses and process rates at the community level. In our model simulation, increasing animal diversity led to an increase in biomass of the animal compartment of the ecosystem despite higher energetic losses of the animal community caused by higher rates of respiration and intraguild predation. Metabolic losses of animals increased proportionately to the gains via consumption on plants. The losses due to intraguild predation increased more strongly in relative terms, but at lower absolute values.

Our results corroborate the hypothesis that high animal diversity should lead to increased intraguild predation, with large species at the top of the food web accumulating more biomass, while small species are losing. However, the high level of intraguild feeding will not *per se* release plants from top–down control and increase plant biomass. Instead, a diverse animal community may be more exploitative without imposing stronger top–down control on plants. The reason for this lack of an effective top–down control is that the plant community also responds to the increased pressure by shifting the community size structure towards larger species. These larger species compensate for higher losses due to herbivore consumption with their lower per unit biomass metabolic rates, which enables them to maintain their levels of biomass as animal diversity increases.

The allometric food web model applied in our study takes body mass as the only differentiating parameter for the particular set of feeding traits and physiological parameters of a species^32^. This simplification of ecological systems assumes that all animal species of equal body mass are equal, in contrast to neutral animal species models, which assume that all species are equal. At the cost of adding only one defining parameter, allometric models provide a much more realistic baseline for the investigation of systemic processes within food webs and may easily be extended to integrate phylogeny and other body-mass independent species traits^47^ as well as the effects of temperature on individual metabolism^33^,^39^,^48^. Most importantly, the model drops the limiting distinction of vertical versus horizontal diversity^8^,^16^. The increase in total intraguild predation with increasing animal diversity is the consequence of the more complete niche coverage since allometry defines the resource range and feeding intensity of consumers on the body-mass axis relative to their own body mass, analogous to classical niche concepts^49^. For plant species and also for smaller and intermediately sized animals, this enhances the likelihood of top–down control by an animal consumer. Thus, allometric feeding rates produce niche complementarity^18^,^41^, a concept that applies when varying horizontal diversity, that is, diversity within the trophic level^8^. Further, the allometric niche differentiation leads to intraguild predation and subsequent indirect effects^16^, or trophic cascades within the food web. Thus, as species number increases the vertical diversity of the community increases^8^, resulting in food webs with a ‘taller' effective trophic height (Fig. 5). The model is consistent with the natural complexity of food webs that are rich in feeding interactions across and within trophic levels^44^.

The model framework further assumes that communities respond dynamically to the quality and quantity of resource supply and consumption in the food web context. In this dynamic process, the biomasses of both animals and plants were allocated towards larger-sized species as animal species number increased. This systematic shift in community body mass structure had strong implications for the effective feeding interactions within the community. The advantage of large animals in species-rich communities led to a stronger limitation of smaller species' populations and enhanced the total direct feeding on plants. However, this increase in feeding was selective for smaller plant species, which resulted in large, slow-growing plant species dominating the community.

The spectrum of simulated communities that we observed at low species richness is relatively wide and heterogeneous, potentially allowing for multiple stable ecosystem states, depending on the feeding traits and food web context of the species present. In contrast, at high animal species richness this spectrum of possible ecosystem states narrows down and becomes more homogeneous with more complementary or redundant species (that is, insurance effects^50^) and a greater probability of including top-predator species of high trophic level (that is, sampling effects^16^,^51^). Thus, increasing diversity consolidates ecosystem function. The sensitivity analysis suggests that the scaling of attack rates with consumer body-mass may also be influential on the relationship between animal diversity and ecosystem function and encourages further exploration. Most importantly, it corroborates previous findings that species-rich communities will be less variable and more predictable in their functioning than communities with few species^52^.

In summary, our food-web simulations indicate that increasing animal diversity, while fostering intraguild predation, does not necessarily release plant biomass from top–down control. Instead, more diverse animal communities favour larger-bodied animal and plant species which balances the effects on plant biomass. We therefore revisit a long-established hypothesis which assumes that increased amounts of intraguild feeding in diverse animal communities will relax the total pressure on plants. This traditional notion originated from static, structural concepts of food webs that neglected compensatory dynamics of complementary species and the resulting complexity of indirect effects. In contrast, the approach of this study offers a concept of body-size regulated community plasticity that reconciles the hypotheses regarding the relationship between animal diversity and plant biomass stock. The mechanisms that have been identified as major drivers of the biodiversity-ecosystem functioning relationship, such as niche complementarity or trophic complexity, are inherent to the allometric food-web model. The limiting assumptions of the model approach also represent important future directions of research that can be added to its flexible model framework to create tailored null hypotheses. Thus, our approach opens new possibilities for future studies of multi-trophic biodiversity and ecosystem functioning. We anticipate that such a mechanistic and dynamic concept of complex, multi-trophic communities is indispensable to overcome the unidirectional cause-consequence approach to biodiversity and ecosystem functioning and to truly understand the dynamic consequences of imminent species loss.

## Methods

### Food-web structure

The model food webs consisted of a basal plant compartment (*P*) and the consumer compartment (‘animals', *A*; Fig. 1). We varied the initial animal species number, *S*_*A*_, from 10 to 100 species with 300 replicates each. The plant community has been standardized to *S*_*P*_=30 species. The log_10_ body mass *m*_*i*_ of any species *i* was drawn from independent uniform distributions within the inclusive limits *μ*_*P*_=(10^0^, 10^6^) for plant species and *μ*_*A*_=(10^2^, 10^12^) for animals, constraining the smallest possible body mass of a plant species to 1 and the largest possible body mass of an animal species to 10^12^. Trophic relations are defined by the success curve of consumers, that is, the probability of a consumer *i* to actually attack and capture an encountered resource *j* (which can be a plant or an animal),


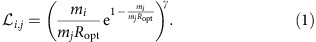


**Figure 2:**
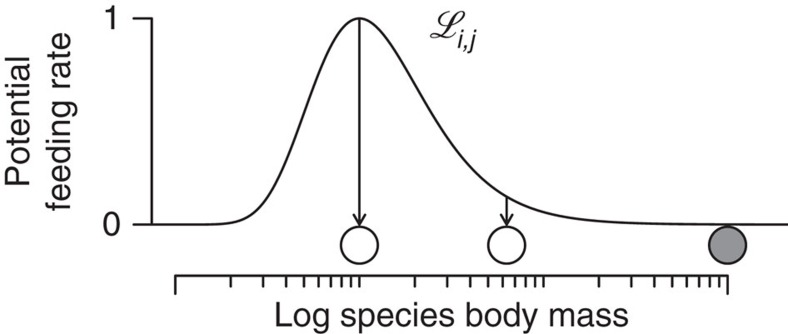
Potential per-capita feeding rate of a consumer species on its resources. The feeding efficiency, 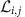 of a consumer (grey circle) on its resources (white circles), is maximized for an energetically optimal resource size relative to its own body mass. Larger or smaller resource species are consumed less efficiently.

**Figure 3:**
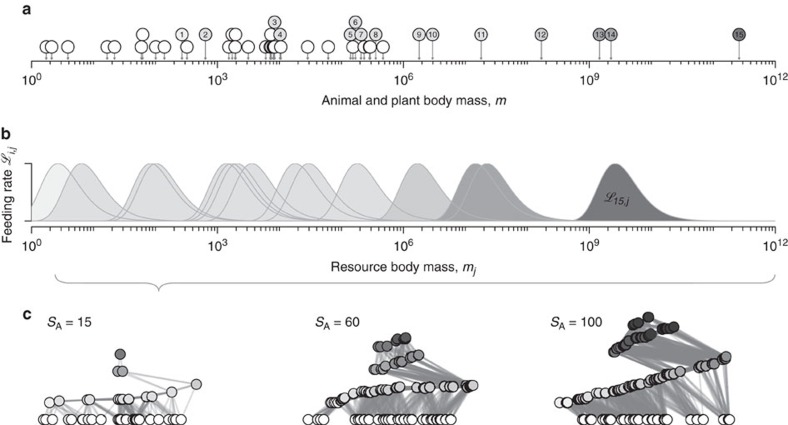
Allometric constraints determine food-web structure. (**a**) Each simulated food web is initiated with *S*_*A*_ animal species (numbered grey circles) on 30 plant species (white circles) of randomly assigned body masses (here: *S*_*A*_=15). (**b**) The potential feeding efficiencies 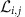 (light curves and areas) of all consumers *i* over the size range *m*_*j*_ describe the functional niche coverage. (**c**) Static network representation of random food webs. A link is drawn if the consumer feeds on resource with 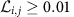 (Methods section). Plant species are ordered by body mass, animal consumers are ordered by the average position of their direct resource species (*x* axis) and by their average trophic level (*y* axis).

**Figure 4:**
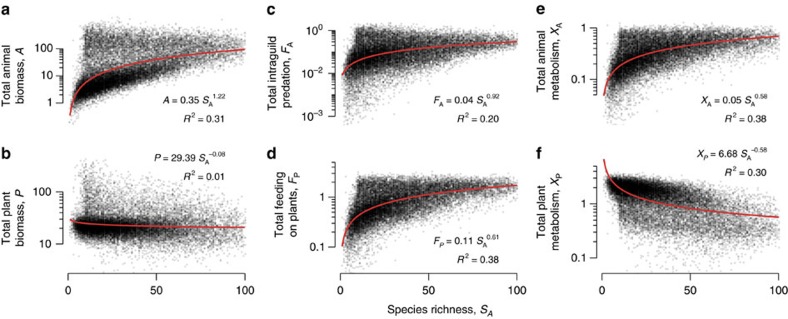
Effect of species richness on ecosystem functions in dynamic communities. Shown are distributions of 21,461 randomly assembled food webs at equilibrium. Increasing species richness increased total animal biomass (**a**) but maintained biomass of the plant community at the same levels (**b**). Intraguild-consumption within the animal community was increased (**c**), as was the consumption on the plant species (**d**). Metabolic losses of animals increased (**e**), while losses of plant species decreased (**f**). Equations and red lines show fitted power-law models (Methods section). Coefficient of determination *R*^2^ indicates goodness of fit.

**Figure 5:**
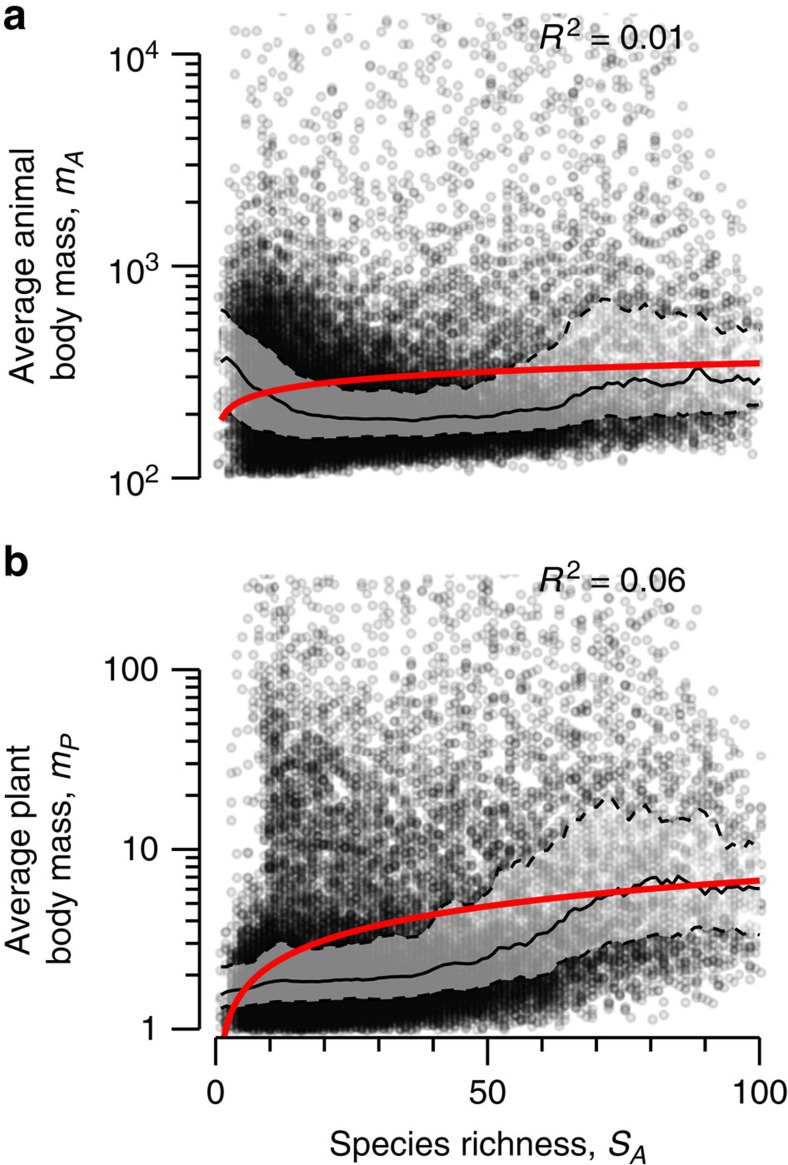
Average individual body mass in response to animal species richness. Observed average individual body mass of (**a**) animals and (**b**) plants. Equations and red lines show fitted power-law models (Methods section); grey line shows median body mass (calculated as median of body mass at each *S*_*A*_±2 with 50%-inner quantile range). Coefficient of determination *R*^2^ indicates goodness of fit. The *y*-axes were truncated to include 95% of simulated food webs.

**Figure 6:**
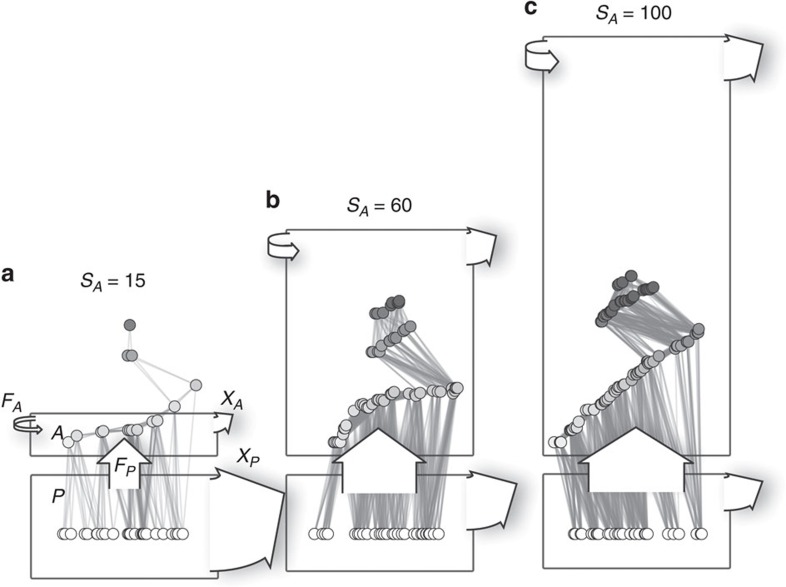
Visualized changes of ecosystem functions at varying animal species richness. Communities visualized for (**a**) 15, (**b**) 60 and (**c**) 90 species (*S*_*A*_). Arrow width at the base is proportional to biomass change per time; Area of boxes is proportional to biomass stocks. While animal biomass, *A*, increased along the gradient due to an increased turnover within the animal community (arrows *F*_*P*_, *F*_*A*_ and *X*_*A*_), biomass of plants, *P*, could be maintained at the same level owing of a slowing down of biomass turnover in the plant community (arrow *X*_*P*_).

It is defined as an asymmetrical hump-shaped curve (Ricker function)^53^ with width *γ*=2, centred around an optimal consumer-resource body-mass ratio *R*_opt_=100 (Fig. 2)^37^. This success curve is subsequently termed the ‘feeding efficiency'. Very weak links with 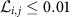 were removed from the model networks, yielding food webs as depicted in Fig. 3c. Note, that in this model we use body mass as the only determinant of a generalist resource choice for both carnivorous and herbivorous feeding. We acknowledge that this simplifying assumption might not reflect the diversity of natural feeding relationships. Especially in terrestrial above-ground food webs, other species traits can be more relevant in determining a feeding link, for example, specialized insect herbivores feeding on large plants may be limited by plant defense or traits rather than size. However, universal body mass constraints on feeding are found in many aquatic and belowground terrestrial habitats^44^. Implementing additional, size-independent constraints on feeding and higher degrees of specialization might be an avenue for future investigations.

### Feeding rates

The allometric model for the rate at which consumer *i* feeds on a resource *j* applies a multi-prey Holling-type functional response with variable Hill-exponent^54^, and includes intra-specific consumer interference (Beddington–DeAngelis type)^32^,^55^,^56^. The feeding rate,


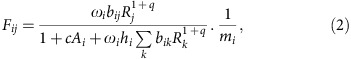


of one unit of biomass of the consumer, *i*, (transformed from per capita feeding rates by dividing by individual body mass, *m*_*i*_) is a function of the biomass density of the consumer, *A*_*i*_, and biomass density of the resource, *R*_*j*_, which can be an animal or a plant species (thus substitute *A*_*j*_ or *P*_*j*_). It includes the resource specific capture coefficient,


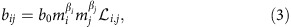


of a consumer species *i* on a resource species *j*, which scales the feeding efficiency 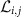 by a power function of consumer and resource body mass, assuming that the rate of encounters between consumer and resource scales with their respective movement speed. Thus, *b*_*ij*_ increases according to a power law with the body masses of consumer (*m*_*i*_) and animal resource (*m*_*j*_)^35^. For each food web replicate, the exponents *β*_*i*_ and *β*_*j*_ were sampled from normal distributions with mean 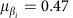, and s.d. 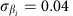, and 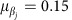 and 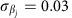, respectively^39^. Since plants do not move, we assumed a constant 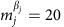 for plant resources. We further assumed a constant *b*_0_=50 for all capture coefficients. The relative consumption rate *ω*_*i*_ accounts for the fact that a consumer has to split its consumption if it has more than one resource species. It thus is defined as *ω*_*i*_=1/(number of resource species of *i*). Further, the feeding rate includes the time lost due to consumer interference *c*, the proportion of time that a consumer spends encountering con-specifics^55^, which is independent of body mass^15^. For each food-web replicate, *c* was drawn from a normal distribution (*μ*_*c*_=0.8, *σ*_*c*_=0.2). The density-dependent change in search efficiency is implemented via the Hill-exponent 1+*q*, which reduces the feeding rate for low resource densities and varies the functional response between classic type II (*q*=0) and type III (*q*=1)^54^,^55^. The value of *q* was drawn for each replicate from a normal distribution (*μ*_*q*_=0.5, *σ*_*q*_=0.2) within the inclusive limits of 0 and 1 (invalid draws were repeated), reflecting that different ecosystems provide specific levels of habitat heterogeneity that reduce feeding at low resource density, for instance by providing refuges^55^. Finally, the handling time,


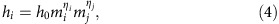


depends on the body mass of the consumer to the power of *η*_*i*_ (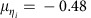, 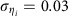) and the body mass of the resource to the power of *η*_*j*_ (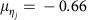, 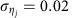), with the scaling constant *h*_0_=0.4 (refs 31, 39).

All exponents were sampled within the exclusive limits of ±3*σ*. Invalid draws were repeated.

### Population dynamics

The model food webs were energetically based on a dynamic nutrient model with two nutrients of different importance supplying the plant community^57^,^58^. On top, a variable number of consumers were feeding on the plant species and among each other as defined by the food-web structure.

The rate of change of the biomass density of an animal species *j* is defined as


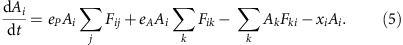


The first-term describes the summed gain by consumption of plant species *j* times the conversion efficiency *e*_*P*_=0.45 typical for herbivory that determines the proportion of biomass of eaten resource that can be converted into own biomass^31^. The second term is identical, but refers to the summed gain by consumption of other animal species *k* times a conversion efficiency *e*_*A*_=0.85 for carnivorous consumption^31^. The third-term sums the mortality due to predation by other animal species *k*. The metabolic demands per unit biomass for animals are defined to scale allometrically with 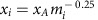 (that is, corresponding to a 34 power-law scaling of per capita metabolic rates)^31^,^58^, using the scaling constant^31^ *x*_*A*_=0.314.

Similarly, the rate of change of the biomass density of any plant species *i* is defined as


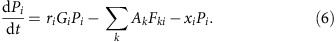


The first-term describes growth due to the uptake of nutrients (see below). The second-term describes mortality due to predation by animals, summed over all consumers *k* of plant species *i*. Finally, each plant species has metabolic demands, 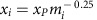, which scale allometrically with its body mass *m*_*i*_, using *x*_*P*_=0.138 as a constant^31^,^58^.

The growth of a plant species is limited by its intrinsic growth rate^59^ 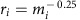 and by the species specific growth factor *G*_*i*_ which is determined dynamically by the concentration of the nutrient 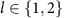 that is most limiting to *i*:


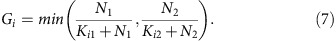


For high nutrient concentrations, the term in the minimum operator approaches 1. The half-saturation densities *K*_*il*_ determine the nutrient uptake efficiency and are assigned randomly for each plant species *i* and each nutrient *l* (uniform distribution within the inclusive limits of 0.1 and 0.2). This model makes plants compete for resources, which is an essential feature of dynamic ecosystem functions, and generates niche differentiation of the plant species^60^, which reduces the risk of competitive exclusion^57^,^58^. The dynamic change of nutrient concentration *N*_*l*_ is defined by


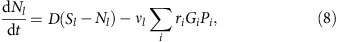


with a global turnover rate *D*=0.25 that determines the rate by which nutrients are refreshed^58^. The supply concentration *S*_*l*_ determines the maximal nutrient level drawn at random from a normal distribution (*μ*_*S*_=10, *σ*_*S*_=2) and is constrained to be larger than 0. The nutrient stock is diminished by the summed uptake by all plants *i*. The loss of a specific nutrient *l* is limited by its relative content in the plant species' biomass (*v*_1_=1, *v*_2_=0.5).

The population dynamics were calculated by integrating the system of differential equations implemented in C using procedures of the SUNDIALS-CVODE solver (backward differentiation formula; absolute and relative error tolerances of 10^−10^)^61^,^62^. Nutrient concentrations *N*_*l*_ were initialized with random values uniformly distributed between *S*_*l*_/2 and *S*_*l*_, animal and plant biomass densities were initialized with random values uniformly distributed between 0 (exclusive) and 10 (inclusive). The food webs were simulated until *t*=150,000 to ensure that stationary dynamics were reached. Species were assumed to be permanently extinct from the food-web once their biomass fell below a threshold, that is, if *A*_*i*_ or *P*_*i*_≤10^−6^ it was immediately set to 0. Replicates that included consumer-free basal species at the end of the simulation time were discarded from the data set (*n*=5,839, corresponding to 21% of the simulations initialized). The uncontrolled growth of such inedible basal species would outcompete other plants and reduce overall species richness drastically, leading to a fundamentally different type of ecosystem^28^. In total, 21,461 valid food webs were simulated.

### Output parameters

The total biomass stocks of the animals, *A*, and the plants, *P*, were calculated as the average of the summed biomasses of all species over an evaluation period of 10,000 time steps after the population dynamics had reached a stationary state. Rates of biomass flow from plants to animals, *F*_*P*_ (herbivory), and among animals (carnivory or intraguild predation), *F*_*A*_, were calculated as average biomass transfer per time step over the same evaluation period. Note, that these flows represent the rate of biomass production by plants and animals, respectively, since we calculated them before accounting for losses due to incomplete assimilation. Total metabolic rates of animals, *X*_*A*_, and plants, *X*_*P*_, were calculated as the sum of the metabolic rates multiplied with the average biomass densities of animal and plant species, respectively.

### Statistical models

The basal and consumer stocks, rates and losses were statistically described as power laws of *S*_*A*_ of the form 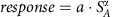. A log–log-transformation yielded the linear model structure of the form *log*(*x*)=*log*(*a*)+*α log*(*S*_*A*_) which was fitted using least squares (using the function lm() in R v3.2.2 (ref. 63)). We report the coefficient of determination, *R*^2^, as a goodness of fit metric for the linear model. For the linear model predicting intraguild predation, replicates with value zero were omitted (*n*=28; 0.1% of all replicates).

### Data availability

All relevant computer codes and simulation results are available online^64^ including the original simulation source code (written in C) as well as the code for the statistical analysis and figure generation (written in R); Code repository on GitHub: https://github.com/fdschneider/schneider_et_al_2016_animaldiversity.

## Additional information

**How to cite this article:** Schneider, F. D. *et al*. Animal diversity and ecosystem functioning in dynamic food webs. *Nat. Commun.* 7:12718 doi: 10.1038/ncomms12718 (2016).

## Supplementary Material

Supplementary Information Supplementary Figures 1-9, Supplementary Methods and Supplementary References.
