# Supplementary material for: Animal diversity and ecosystem functioning in dynamic food webs
Source: Nat Commun. 2016 Oct 5;7:12718. doi: 10.1038/ncomms12718 (PMC5059466; doi:10.1038/ncomms12718)
Supplement: Supplementary Information — Supplementary Figures 1-9, Supplementary Methods and Supplementary References. [file ncomms12718-s1.pdf]

Supplementary Figures

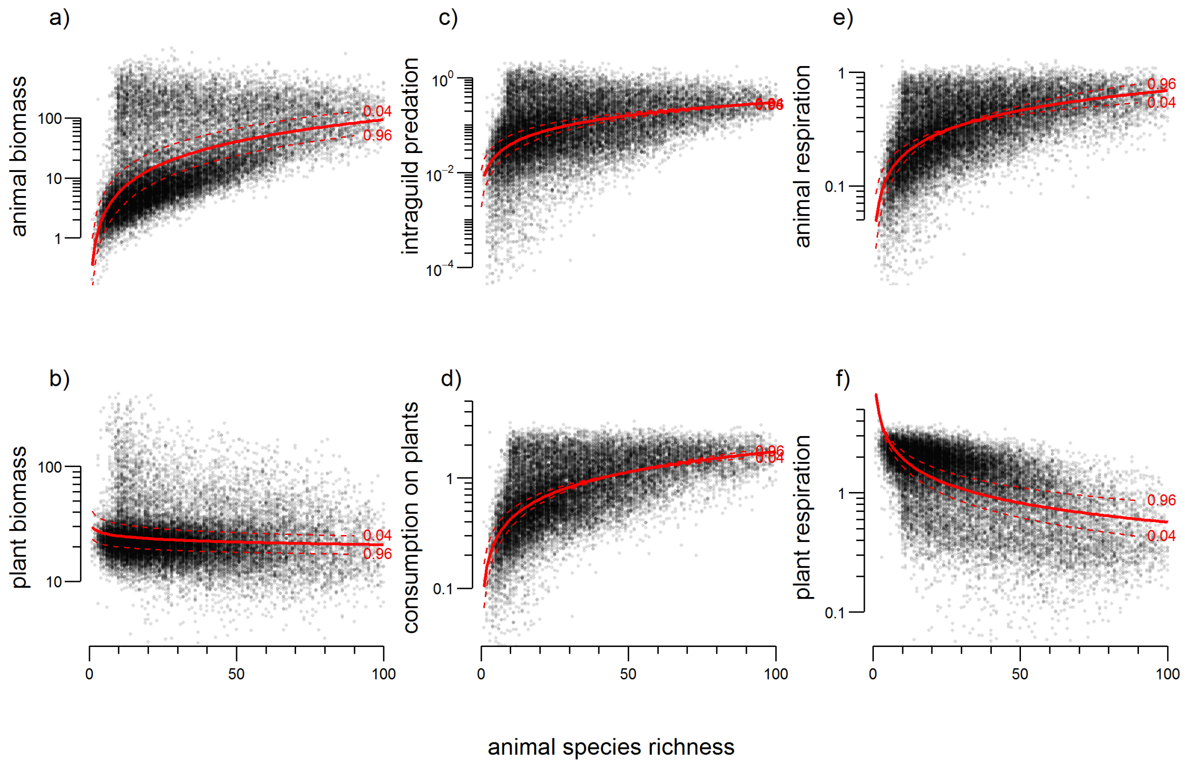

Supplementary Figure 1 – Effect of Hill-exponent of feeding rate on relationship between animal species richness and ecosystem functions. Influence of Hill-exponent, q, on steady state (a) animal biomass, (b) plant biomass, (c) intraguild feeding, (d) consumption on plants, (e) animal respiration and (d) plant respiration. Solid line is linear model of average q; dashed lines are covering model predictions for 99% of variation in q (99% interquantile range: 0.04 – 0.96).

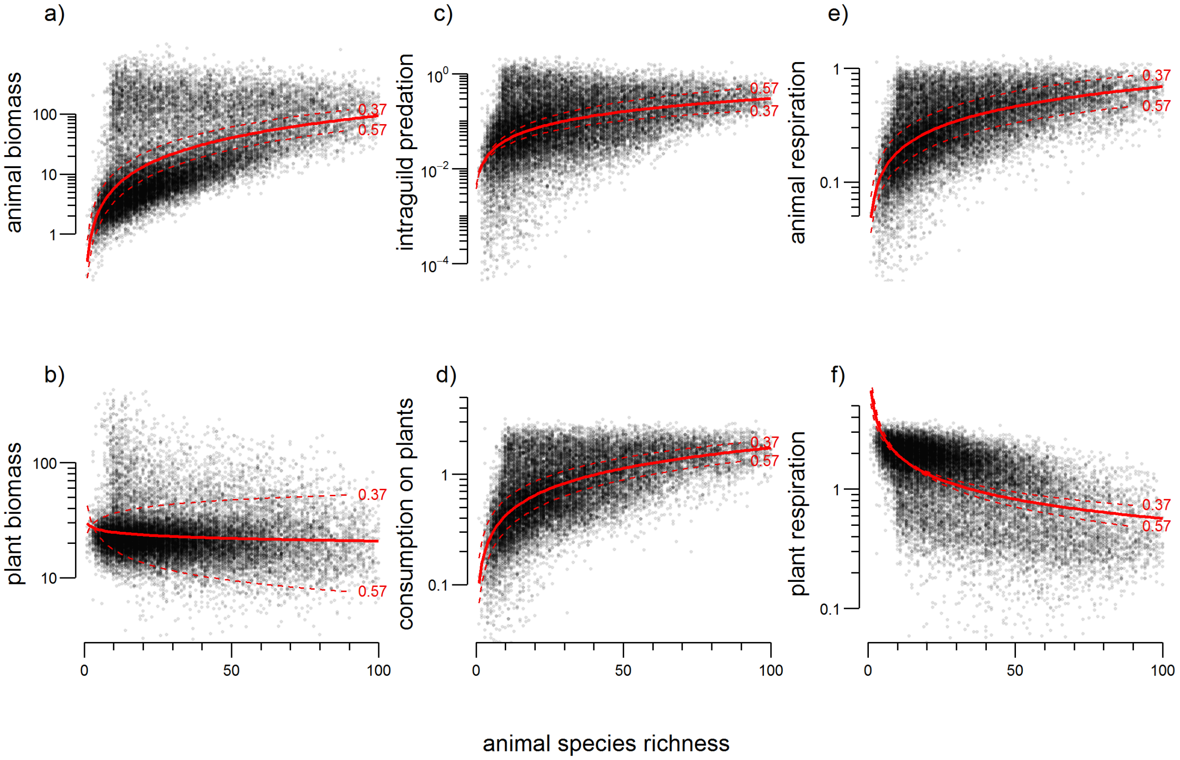

Supplementary Figure 2 – Effect of consumer body mass in attack rate of feeding rate on relationship between animal species richness and ecosystem functions. Influence of Scaling exponent, β_i, on steady state (a) animal biomass, (b) plant biomass, (c) intraguild feeding, (d) consumption on plants, (e) animal respiration and (d) plant respiration. Solid line is linear model of average β_i; dashed lines are covering model predictions for 99% of variation in β_i (99% interquantile range: 0.37 – 0.57).

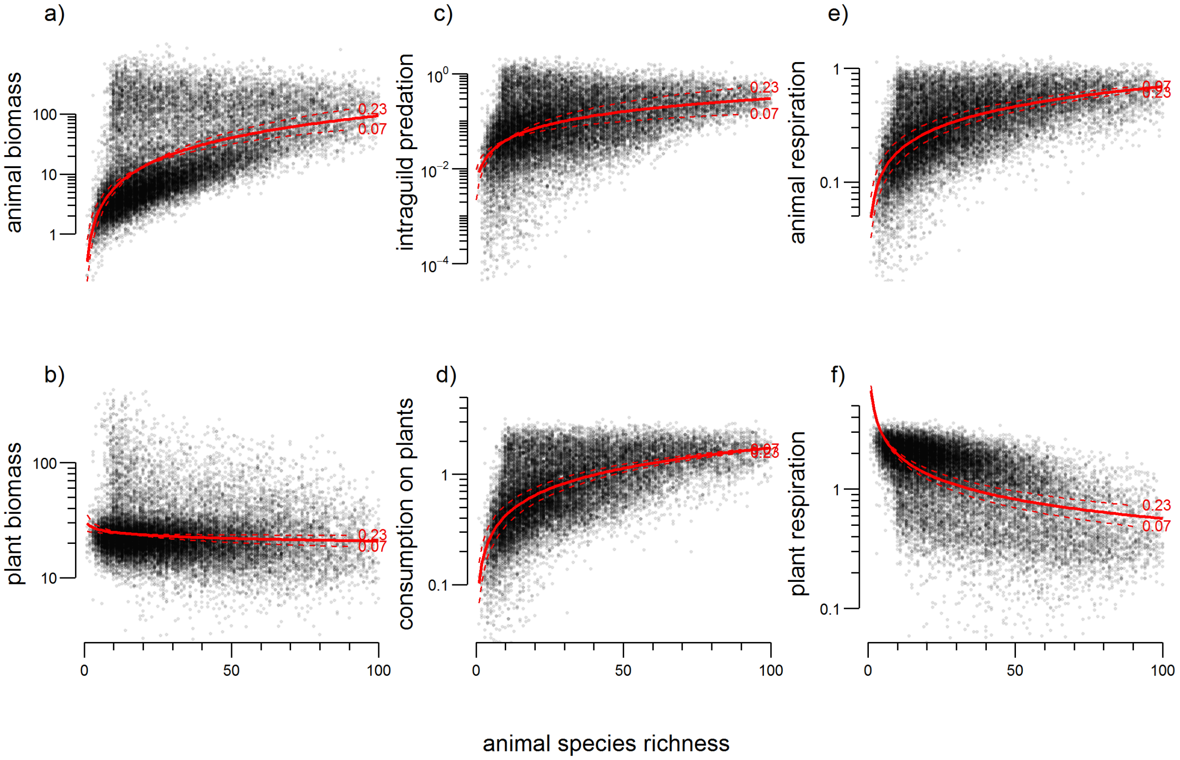

Supplementary Figure 3 – Effect of resource body mass in attack rate of feeding rate on relationship between animal species richness and ecosystem functions. Influence of Scaling exponent, β_j, on steady state (a) animal biomass, (b) plant biomass, (c) intraguild feeding, (d) consumption on plants, (e) animal respiration and (d) plant respiration. Solid line is linear model of average β_j; dashed lines are covering model predictions for 99% of variation in β_j (99% interquantile range: 0.07 – 0.23).

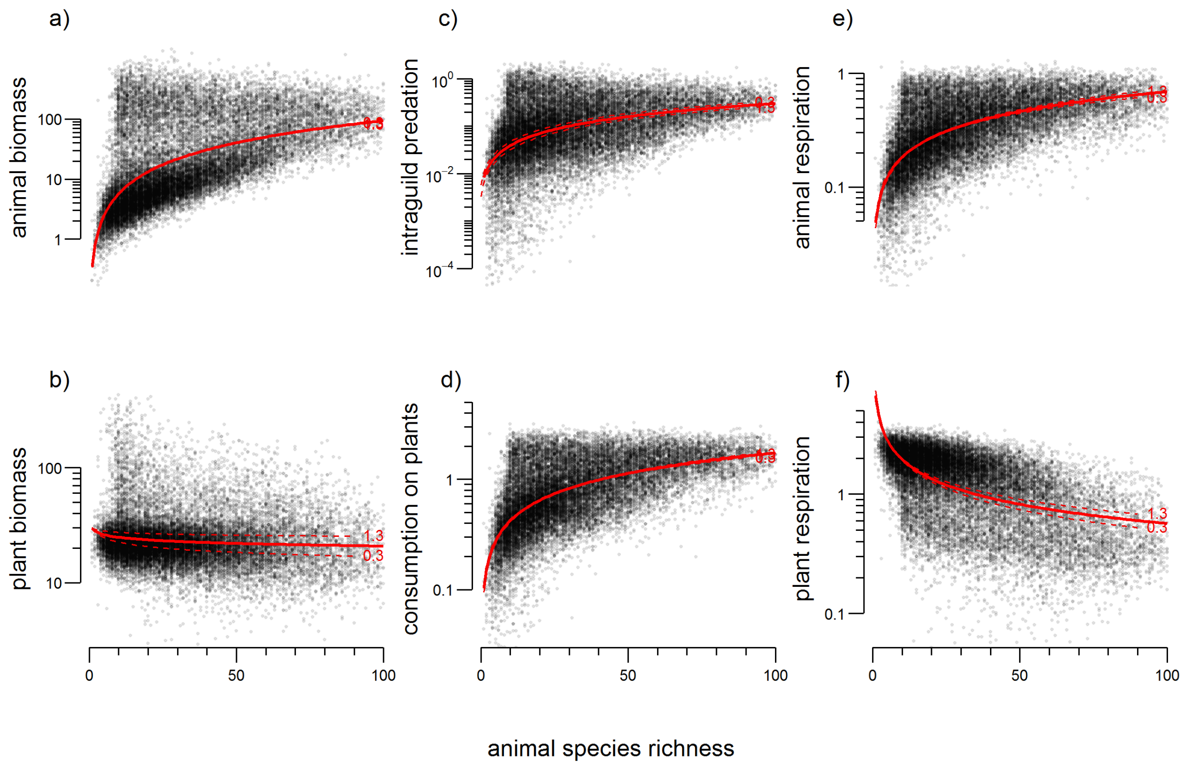

Supplementary Figure 4 – Effect of predator interference in attack rate of feeding rate on relationship between animal species richness and ecosystem functions. Influence of interference coefficient, c, on steady state (a) animal biomass, (b) plant biomass, (c) intraguild feeding, (d) consumption on plants, (e) animal respiration and (d) plant respiration. Solid line is linear model of average c; dashed lines are covering model predictions for 99% of variation in c (99% interquantile range: 0.3 – 1.3).

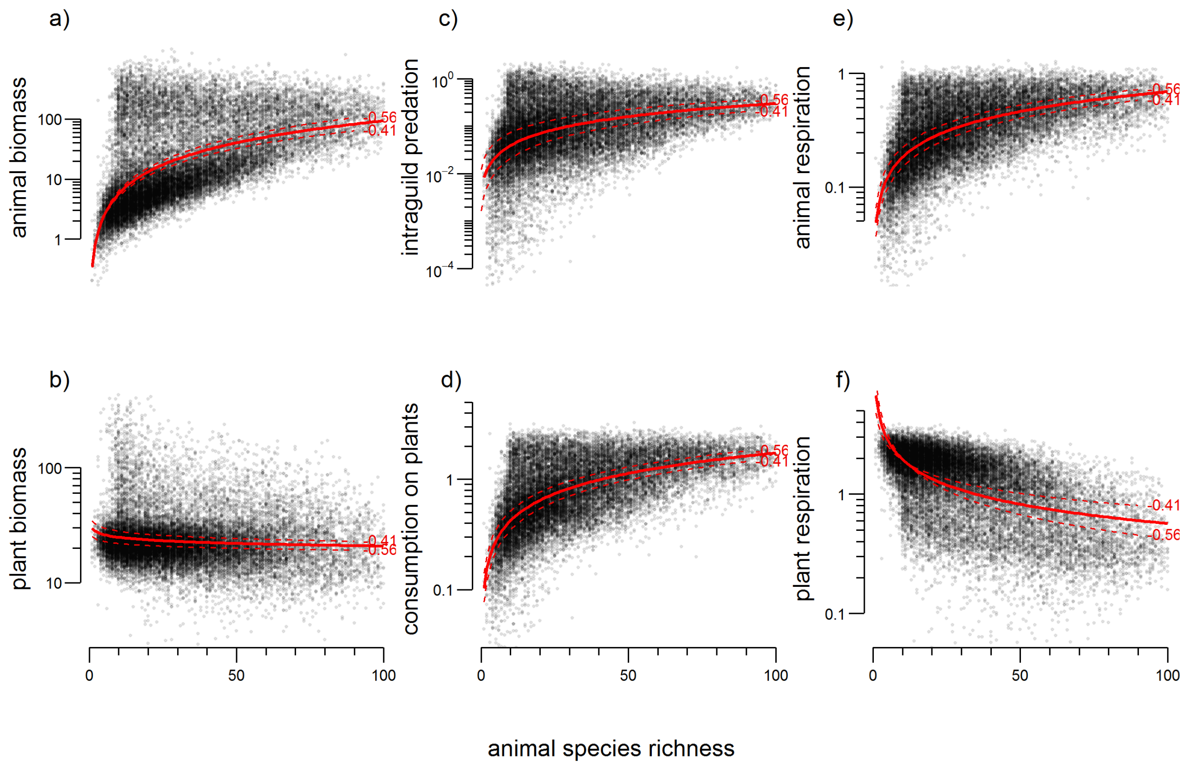

Supplementary Figure 5 – Effect of consumer body mass scaling in handling time of feeding rate on relationship between animal species richness and ecosystem functions. Influence of scaling exponent, η_i, on steady state (a) animal biomass, (b) plant biomass, (c) intraguild feeding, (d) consumption on plants, (e) animal respiration and (d) plant respiration. Solid line is linear model of average η_i; dashed lines are covering model predictions for 99% of variation in η_i (99% interquantile range: −0.56 – −0.41).

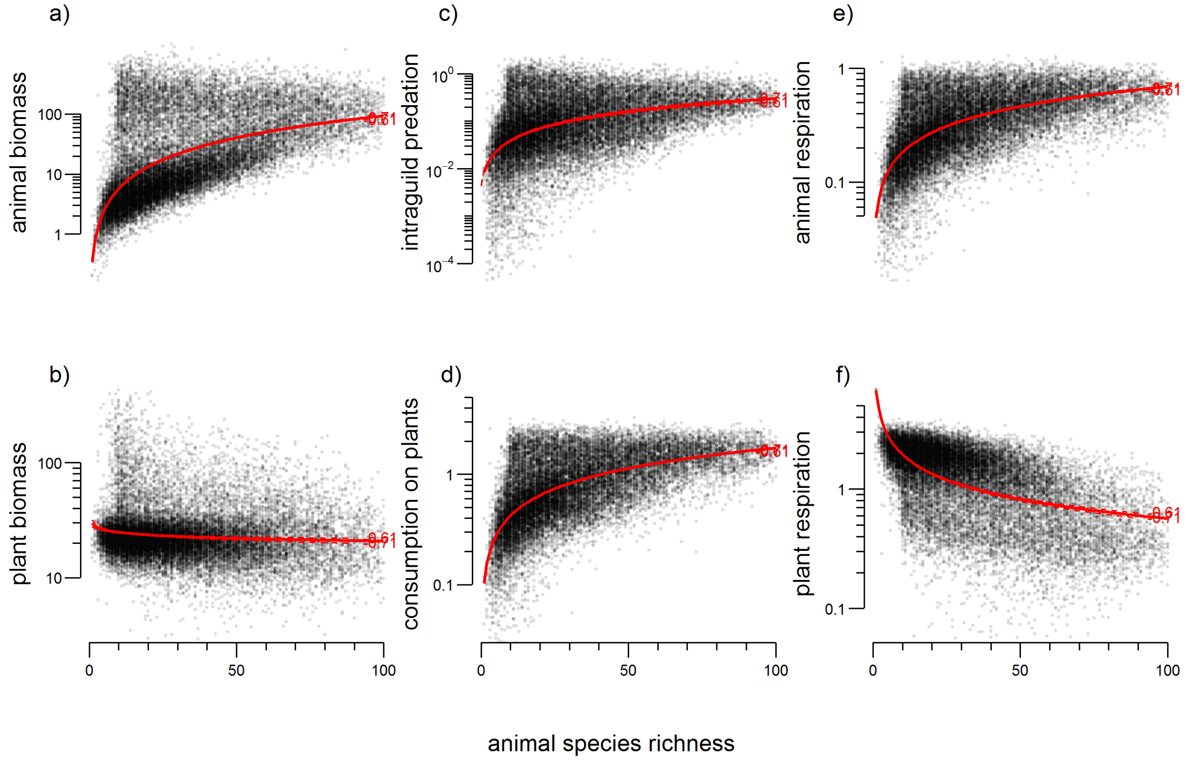

Supplementary Figure 6 – Effect of resource body mass scaling in handling time of feeding rate on relationship between animal species richness and ecosystem functions. Influence of scaling exponent, η_j, on steady state (a) animal biomass, (b) plant biomass, (c) intraguild feeding, (d) consumption on plants, (e) animal respiration and (d) plant respiration. Solid line is linear model of average η_j; dashed lines are covering model predictions for 99% of variation in η_j (99% interquantile range: −0.71 – −0.61).

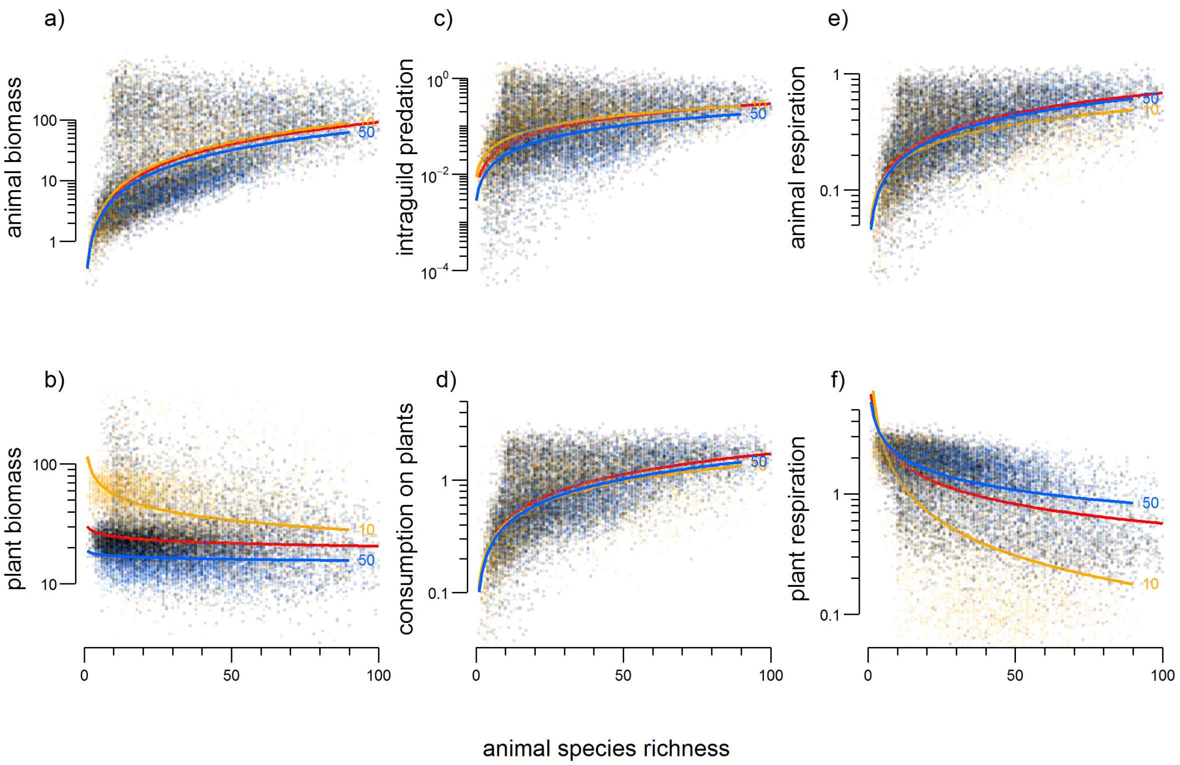

Supplementary Figure 7 – Effect of number of plant species richness on relationship between animal species richness and ecosystem functions. Influence of the number of initial plant species , S_P, on steady state (a) animal biomass, (b) plant biomass, (c) intraguild feeding, (d) consumption on plants, (e) animal respiration and (d) plant respiration. Yellow and blue lines show alternative simulations with low ($S_P = 10$) and high ($S_P = 50$) initial plant species richness, respectively.

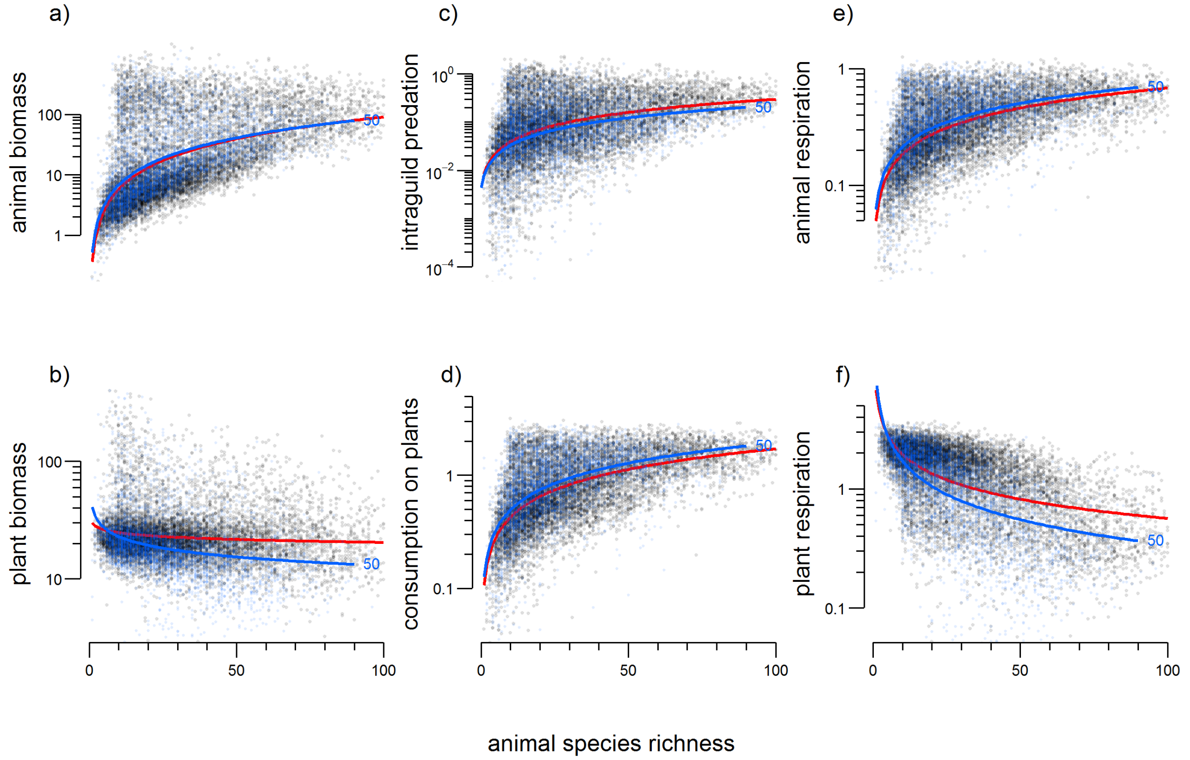

Supplementary Figure 8 – Effect of having a pronounced trophic structure on the relationship between animal species richness and ecosystem functions. Effect of having 50% strict herbivores within the animal community on steady state (a) animal biomass, (b) plant biomass, (c) intraguild feeding, (d) consumption on plants, (e) animal respiration and (d) plant respiration. Blue line show an alternative simulation with 50% of animals strictly limited to herbivorous consumption.

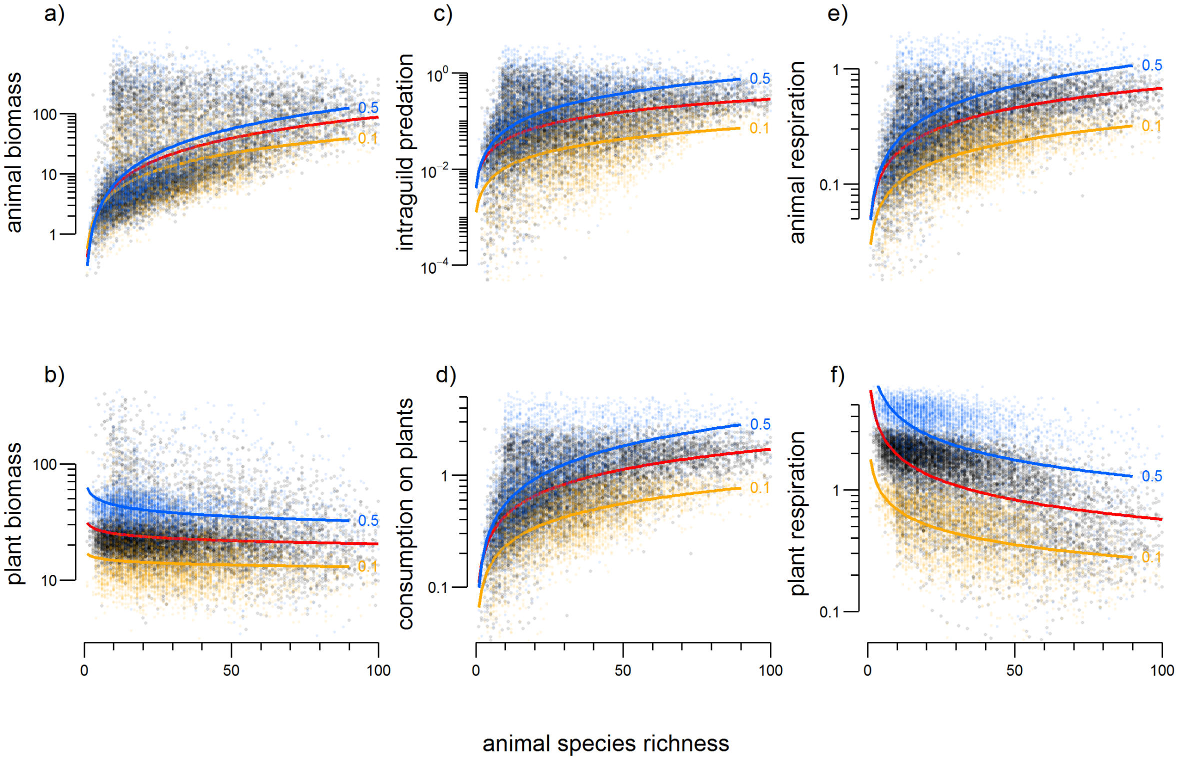

Supplementary Figure 9 – Effect of nutrient supply on relationship between animal species richness and ecosystem functions. Influence of nutrient turnover rate, D, on steady state (a) animal biomass, (b) plant biomass, (c) intraguild feeding, (d) consumption on plants, (e) animal respiration and (d) plant respiration. Yellow and blue lines show alternative simulations with low ($D = 0.1$) and high ($D = 0.5$) turnover rates, respectively.

Supplementary Methods

We performed a series of complementary analyses to test if the conclusions we draw in the paper are robust to changes in the model parameters.

Stochastic parameters

In the article, we argue for the parameters we applied for allometric constants or optimal body mass scaling based on empirical evidence, but we included stochastic noise to cover uncertainties about the precise values. For the model simulations, we sampled all scaling exponents from normal distributions within the inclusive limits of $\pm 3\sigma$. Similarly, parameters of the functional response (Hill-coefficient and predator interference) were drawn from a normal distribution. Here, we analyse how changes in those parameter values affect the resulting relationship between species richness and the ecosystem function, y, by fitting linear models that contain the parameter in question, x, as well as it's interaction term with S_A.

$$\log y = \log(a) + \alpha \log(S_A) + \beta X + \gamma X \log(S_a). \quad (1)$$

We illustrate the relative importance of those parameters by reproducing figure 4 from the manuscript with the linear model predictions for lowest and highest parameter values. The lowest and highest values are defined by the 99%-interquantile range of the parameter, i.e. the parameter threshold values that incorporate 99% of all replicates. The full code for this sensitivity analysis is available online at https://github.com/fdschneider/schneider_et_al_2016_animaldiversity.

Hill-exponent. The Hill-exponent of the functional response varies around 0.5 with a standard deviation of 0.2 within the inclusive limits of 0 and 1. This is based on the frequent observation of moderate type-III functional responses in natural systems[1,2]. For reasons of simplicity, we assume a unified type of functional response in each habitat. This reflects that the reduction of capture coefficients at very low resource densities depends on the habitat heterogeneity. In natural ecosystems, the shape of the sigmoidal functional response will differ among taxa, but is systematically bound to body size ratio[3].

We find that the value of the Hill-exponent does not alter the relationship between functional diversity and ecosystem functions qualitatively (Supplementary Fig. 1). A strong sigmoidal shape of functional response (i.e. high Hill-exponents) reduces total animal biomass. This reduction in animal biomass propagates to plant biomass accompanied by an increase in plant respiration (Supplementary Fig. 1).

Capture coefficient. The allometric feeding rate model includes a definition of the capture coefficient, b_{ij} that scales the feeding efficiency $\mathscr{L}_{i,j}$ with the body mass of consumer, m_i, and the body mass of the resource, m_j, following power-law relationships. For each food web, the exponent of consumer body mass was drawn from a normal distribution with mean $\mu_{\beta_i} = 0.47$, and standard deviation $\sigma_{\beta_i} = 0.04$. The exponent of resource body mass was drawn from a normal distribution with $\mu_{\beta_j} = 0.15$ and $\sigma_{\beta_j} = 0.03$. These means were estimated from empirically observed values[1]. For plant resources, the capture coefficient did not scale with resource body mass and the scaling with resource body mass was subsituted by a constant $m_j^{\beta_j} = 20$.

The exponent of consumer body mass is influental in altering the effect of animal species richness on total plant biomass from positive at low values and negative at high values (Supplementary Fig. 2). Other ecosystem functions responded only weakly to changes in the exponent of consumer body mass. The influence of the exponent of resource body mass was weak (Supplementary Fig. 3) and did not alter the relationship between animal species richness and ecosystem function qualitatively.

Consumer interference. We assumed that consumers of the same species engaged in non-fatal interactions that reduced their time dedicated to foraging as their own density increased. The value of predator interference was drawn from a normal distribution with $\mu_c = 0.8$ and $\sigma_c = 0.2$. The effect of predator interference is negligible and does not alter the relationship between animal species richness and ecosystem function (Supplementary Fig. 4).

Handling time. The time a consumer spends on handling one prey item is a power-law of both consumer body mass, m_i, and resource body mass, m_j with exponents η_i and η_j, respectively, drawn from normal distributions ($\mu_{\eta_i} = -0.48$, $\sigma_{\eta_i} = 0.03$; $\mu_{\eta_j} = -0.66$, $\sigma_{\eta_j} = 0.02$). Neither of the two exponents alters the relationship between animal species richness and ecosystem function (Supplementary Fig. 5 & 6).

Structural parameters

Other parameters of the model were of structural character, such as the number of basal plant species or the type and amount of nutrient supply. To show how changes in those parameters alter the relationship of biodiversity and ecosystem function we performed additional simulations. For each alternative parameter value, we ran 60 replicates of each step along the gradient of 10 to 100 animal species and fitted independent linear models for each simulation on log-log transformed data. We compare these data and linear models to a subset of 60 replicates per initial animal species richness from the original model.

Number basal plant species. In the default model, we varied the diversity within the animal community whereas the basal trophic level of plants was initialized with a standardized species richness of 30. This methodology was chosen to ensure that differences in plant productivity such as changes in the total carrying capacity of all basal species cannot confound our results. Allowing extinctions during the population dynamics might further reduce this number in some of the food webs. In the model, the animal species number varied from 10 to 100, which causes very different ratios between the number of animal and plant species. The number of plant species might have an impact on the number of animal species that the ecosystem can support and the distribution of the functional diversity along the body mass axis. Thus, in this analysis we simulated food webs with 10 and 50 plant species to visualise the effect of the initial number of plant species for the relationship between animal diversity and ecosystem functioning.

Increased plant species richness did not alter the patterns qualitatively, but reduced the level of plant biomass while increasing plant respiration rates. Strong effects were visible for low plant species richness which led to reduced plant respiration at high animal diversity and a negative relationship of animal diversity and plant biomass (Supplementary Fig. 7). We caution, however, that the model dynamics would also affect the selective survival of animal species in communities with few plants spaced out along the body mass axis. Thus, the animal community is less likely to comprise multiple trophic levels. This illustrates how the reduction of plant diversity propagates up the food chain.

Strict herbivory. The foodwebs constructed by our model assume that most animals are omnivorous feeders that include both plants and animals in their resource spectrum. In nature, many strict herbivores are forming the level of primary consumers, whithout supplementary energy uptake via other pathways. To test how this simplification affects the results of our study, we simulated foodwebs with 50% strict herbivores among the primary consumers, thus strengthening the direct flow of energy into the animal compartment.

This did reduce plant biomass at high levels of animal species richness, as well as the losses due to plant respiration (Supplementary Fig. 8). The top-down supression seems to work better in this case.

Nutrient supply. To see how bottom-up supply alter the relationship between functional diversity and ecosystem function, we ran the simulation with higher and lower rates of nutrient turnover ($D = 0.1$ and $D = 0.5$, respectively). This changed the absolute values of biomass stocks and consumption and respiration rates, but did not alter the relative change of those values along the gradient of functional diversity (Supplementary Fig. 9).

Supplementary References

1.Rall, B. C. *et al.* Universal temperature and body-mass scaling of feeding rates. *Philosophical Transactions of the Royal Society B: Biological Sciences* **367,** 2923–2934 (2012).

2.Englund, G., Öhlund, G., Hein, C. L. & Diehl, S. Temperature dependence of the functional response. *Ecology Letters* **14,** 914–921 (2011).

3.Kalinkat, G. *et al.* Body masses, functional responses and predator–prey stability. *Ecology Letters* **16,** 1126–1134 (2013).
